# Medical Mycology Education in Brazil: A Cross-Sectional Survey of Medical Schools

**DOI:** 10.1093/ofid/ofaf540

**Published:** 2025-09-02

**Authors:** Alessandro C Pasqualotto, Valerio R Aquino, Diego R Falci, Cecilia B Severo, Melissa O Xavier, Eduardo N Trindade

**Affiliations:** Infectious Diseases Service, Santa Casa de Porto Alegre, Porto Alegre, Brazil; Post-Graduation Program in Pathology, Federal University of Health Sciences of Porto Alegre (UFCSPA), Porto Alegre, Brazil; Infectious Diseases Service, Hospital de Clinicas de Porto Alegre (HCPA), Porto Alegre, Brazil; Infectious Diseases Service, Hospital de Clinicas de Porto Alegre (HCPA), Porto Alegre, Brazil; Internal Medicine and Diagnostics Department, Pontifical Catholic University of Rio Grande do Sul (PUCRS), Porto Alegre, Brazil; Post-Graduation Program in Pathology, Federal University of Health Sciences of Porto Alegre (UFCSPA), Porto Alegre, Brazil; Post-Graduation Program in Health Sciences, Federal University of Rio Grande (FURG), Rio Grande, Brazil; President's Office, Regional Medical Council of the State of Rio Grande do Sul (CREMERS), Porto Alegre, Brazil

**Keywords:** fungal infections, infectious diseases, medical education, medical mycology, undergraduate curriculum

## Abstract

**Background:**

Despite the high burden of fungal infections in Brazil, little is known about how medical mycology is taught in Brazilian medical schools. Adequate education is essential to improve clinical recognition, diagnosis, and treatment of fungal diseases.

**Objectives:**

To assess the structure, content, and challenges of medical mycology education across medical schools in Rio Grande do Sul, Brazil.

**Methods:**

A cross-sectional survey was conducted among all 21 medical schools in the state. Nineteen schools (90.5%) responded to a structured questionnaire addressing curricula, infrastructure, faculty training, and perceived barriers.

**Results:**

All responding institutions include medical mycology in their curricula, though none offer it as a stand-alone subject. Teaching is typically embedded within microbiology (89.5%) and infectious diseases (57.9%). Only 21.1% dedicate over 40 h to the topic. While most schools address superficial (94.7%), systemic (94.7%), subcutaneous (89.5%), and opportunistic (94.7%) mycoses, practical diagnostic exercises are limited. Only 52.6% provide hands-on diagnostic training, and 42.1% have a dedicated mycology lab. Most instructors have doctoral degrees (73.7%), but only 10.5% specialized in mycology. Student evaluation and curricular updates are infrequent, and institutional support for mycology education remains constrained.

**Conclusions:**

Mycology teaching in southern Brazil is widespread but insufficiently structured. Although the state has a strong historical tradition in fungal disease research, significant gaps remain in faculty expertise, infrastructure, and curricular innovation. Targeted investment in laboratory training and faculty development is urgently needed to strengthen clinical education preparedness.

Fungal diseases represent a significant global health concern, particularly among high-risk populations such as immunocompromised individuals, patients with diabetes, and those using invasive medical devices. It is estimated that over 1.5 million people die annually from severe fungal infections such as aspergillosis, candidiasis, and cryptococcosis, with mortality rates often exceeding 50% in critically ill patients [1, 2]. In Brazil, invasive fungal infections are highly prevalent in intensive care units, with associated mortality rates ranging from 30% to 60% [3, 4]. These infections also impose a substantial economic burden due to prolonged therapies, repeated hospitalizations, and the need for advanced diagnostics [5].

Despite their impact, fungal diseases remain under-recognized and neglected within public health systems. Many of these infections are not reportable, resulting in underestimation of their true burden and limiting the development of effective surveillance and control strategies [2, 6]. In Brazil, the lack of official data hampers policy-making and delays investments in diagnostic infrastructure and clinical training [7].

Educationally, the situation is equally concerning. There is a notable absence of published studies examining how medical mycology is taught in Brazilian medical schools. The lack of structured curricula may contribute to inadequate physician preparedness, leading to diagnostic delays and suboptimal patient care [8, 9]. To address this knowledge gap, we evaluated the current status of medical mycology education in medical schools across the state of Rio Grande do Sul, Brazil—a state that currently hosts 21 medical programs.

## METHODS

We conducted a descriptive, cross-sectional study involving all 21 medical schools located in the state of Rio Grande do Sul, southern Brazil. Both public and private institutions were included. A structured, web-based questionnaire was developed by a multidisciplinary panel of experts in medical mycology and medical education, and pretested for clarity and relevance. The survey comprised 14 thematic sections, covering curriculum structure, faculty expertise, teaching methodologies, infrastructure, and perceived barriers in the teaching of medical mycology.

The questionnaire was distributed electronically to course coordinators of all medical schools. Respondents were encouraged to consult with faculty members involved in mycology instruction when completing the survey. Data collection occurred over a 30-day period, with weekly email reminders to increase participation.

Quantitative responses were analyzed using descriptive statistics (frequencies, means, and standard deviations). Categorical variables were compared using the χ^2^ or Fisher's exact test, while continuous variables were analyzed using Student's *t*-test or the Mann–Whitney *U* test, based on data distribution. Open-ended responses were subjected to thematic content analysis. A *P*-value < .05 was considered statistically significant.

The study was approved by the Institutional Review Board of the Federal University of Health Sciences of Porto Alegre (UFCSPA). Participation was voluntary and anonymized, with all data treated confidentially.

## RESULTS

Of the 21 medical schools contacted, 19 participated in the study, yielding a response rate of 90.5%. Among the participating institutions, 7 (36.8%) were public and 12 (63.2%) were private. A total of 6 schools (31.6%) were founded before 1979, 7 (36.8%) between 2000 and 2019, and 6 (31.6%) from 2020 onwards. Regarding student intake, 9 institutions (47.4%) reported enrolling between 51 and 100 students per year, 5 (26.3%) had cohorts of 101–150 students, and 5 (26.3%) admitted more than 150 students annually. All schools reported affiliation with a teaching hospital, with 6 institutions (31.6%) having hospitals with fewer than 200 beds, 13 (68.4%) with 201–500 beds, and none with over 500.

As for graduate output, 5 institutions (26.3%) have already graduated more than 500 students, while 7 (36.8%) have fewer than 100 graduates, reflecting their recent establishment. Seven programs (36.8%) fall between these extremes, with 100–500 graduates.

All 19 respondent institutions confirmed that medical mycology is taught as part of the curriculum; however, none offer it as a stand-alone subject. Instead, mycology is integrated within other disciplines: in 17 schools (89.5%) it is taught within microbiology, in 11 (57.9%) within infectious diseases, and in 6 (31.6%) within parasitology. Only 4 institutions (21.1%) dedicate more than 40 total hours to the topic, while 5 (26.3%) report fewer than 10 h of instruction. In 15 schools (78.9%), mycology content is concentrated in the early years of the medical program. This integration reflects a broader curricular approach, which is common in Brazil, where bacteriology and parasitology are also typically taught within microbiology or infectious disease modules.

Regarding teaching methodologies, lectures are universally employed (100%), laboratory sessions are offered in 13 schools (68.4%), and clinical case-based learning is used in 11 (57.9%). Despite this, only 10 schools (52.6%) include practical activities specific to fungal diagnostics, and just 8 (42.1%) report having a dedicated mycology laboratory. A fungal culture collection is maintained by 9 institutions (47.4%), but only 5 (26.3%) have designated personnel responsible for its upkeep. Use of digital tools or simulation technologies is reported in only 2 programs (10.5%). Training on clinical recognition of fungal infections is present in 15 institutions (78.9%). However, fewer than half (9 institutions, 47.4%) offer hands-on instruction in laboratory techniques for fungal identification.

In terms of content, most institutions teach multiple categories of fungal infections: superficial mycoses are covered in 18 programs (94.7%), subcutaneous mycoses in 17 (89.5%), systemic mycoses in 18 (94.7%), and opportunistic mycoses in 18 (94.7%). However, antifungal pharmacology and therapeutic strategies are included in only 13 programs (68.4%).

Evaluation strategies rely heavily on written tests (used in 17 schools, 89.5%), and only 10 (52.6%) include clinical case-based assessments. Student feedback on mycology teaching is collected in 8 programs (42.1%). Thirteen institutions (68.4%) have never updated their mycology content, and 16 (84.2%) do not reference national or international clinical guidelines in curricular planning.

Faculty involved in teaching is primarily physicians (16 institutions, 84.2%) or pharmacist-biochemists (12 institutions, 63.2%). Regarding academic qualifications, 14 schools (73.7%) reported that their mycology instructors hold doctoral degrees (PhD), and 5 (26.3%) have faculty with postdoctoral training. However, only 2 institutions (10.5%) indicated that these credentials are specifically in medical mycology or related fields.

Research opportunities in the area are available in 13 institutions (68.4%), but extracurricular activities such as seminars or advanced courses in mycology are rare, reported by only 2 institutions (10.5%). The main barriers cited to expanding mycology education include limited laboratory infrastructure (6 institutions, 31.6%) and lack of funding (6 institutions, 31.6%). Finally, 17 institutions (89.5%) report no institutional plans to expand or enhance medical mycology teaching, and 14 (73.7%) lack partnerships with reference laboratories or hospitals specializing in fungal diseases.

## DISCUSSION

This study provides the first comprehensive overview of how medical mycology is taught in undergraduate medical programs in southern Brazil, highlighting substantial variability in content delivery, infrastructure, faculty training, and institutional priorities. Although all 19 participating medical schools report inclusion of mycology within their curricula, the absence of dedicated courses and limited instructional hours reflect a fragmented and marginalized approach of fungal disease education. While geographically limited to southern Brazil, this study represents the first structured assessment of medical mycology education in Latin America and may serve as a replicable model for similar evaluations in other regions.

Importantly, the study achieved participation from 19 of the 21 medical schools in the state of Rio Grande do Sul, corresponding to 90.5% coverage. This high response rate confers strong representativeness to the findings and ensures that the data presented here are a reliable reflection of the current state of medical mycology education in the region.

Our findings are consistent with prior concerns raised in the literature about the underrepresentation of fungal diseases in medical training worldwide, particularly in low- and middle-income countries [1, 2]. While Brazil faces a high burden of invasive fungal infections—especially among critically ill patients, where mortality can exceed 50% [3]—our data suggest that future physicians are often insufficiently prepared to recognize, diagnose, and manage these conditions. Only 21.1% of the schools dedicate more than 40 h to medical mycology, and the majority (78.9%) concentrates teaching in the early years, when clinical context is often lacking.

A major concern is the limited availability of practical training in fungal diagnostics. Although laboratory sessions are offered in most institutions (68.4%), only 52.6% provide specific hands-on activities related to fungal identification. The lack of specialized infrastructure—including mycology laboratories and curated culture collections—was noted in over half of the institutions, which is in line with the current challenges in Brazil and Latin America [10]. These deficiencies may contribute to delays in diagnosis and suboptimal clinical management, as previously reported in Brazilian hospitals.

Faculty qualifications also reveal important limitations. Although most instructors hold doctoral degrees, only a small proportion (10.5%) specifically trained in medical mycology. This may contribute to the outdated nature of curricula in many institutions, with 68.4% reporting that their mycology content has never been revised and 84.2% failing to incorporate contemporary clinical guidelines. The scarcity of postgraduate specialization in this field highlights a broader structural deficiency in fungal disease education and research capacity. Another important limitation is that the study did not evaluate the perceptions or competencies of recent medical school graduates. While their insights could provide valuable information on the long-term impact of mycology training, including this population would have required a systematic approach and a substantially longer data collection period, delaying dissemination. Future studies are planned to incorporate alumni perspectives.

Content-wise, most schools appropriately cover superficial (94.7%), subcutaneous (89.5%), systemic (94.7%), and opportunistic (94.7%) mycoses. However, gaps remain in the teaching of antifungal pharmacology (included in only 68.4% of programs) and clinical mycology diagnostics. Only 42.1% of programs include case-based evaluations, and student feedback is infrequently collected, suggesting that pedagogy remains passive and centered on rote memorization.

The lack of institutional investment is particularly concerning: 89.5% of schools reported no plans to expand medical mycology instruction, and 73.7% lack partnerships with specialized reference centers. In a country with a growing incidence of invasive fungal diseases, this disconnect between epidemiological need and curricular emphasis is striking. Educational strategies must evolve to include experiential learning, integration of diagnostic algorithms, and exposure to clinical cases—especially given the increasing availability of point-of-care diagnostics and molecular tools.

Finally, the low rate of extracurricular engagement (reported in only 10.5% of institutions) and limited student involvement in research (31.6%) suggest missed opportunities to stimulate interest in the field and develop future expertise. Mycology remains a neglected area within the broader spectrum of infectious disease education, mirroring its underprioritization in public health policy.

Historically, medical mycology in Brazil has been shaped by two major academic traditions: the São Paulo school led by Professor Carlos da Silva Lacaz, and the Rio Grande do Sul school, founded by Professor Alberto Thomaz Londero, whose work was carried forward by his distinguished disciple, Professor Luiz Carlos Severo. The contributions of Drs. Londero and Severo have had a profound and lasting impact on the teaching and dissemination of mycology in southern Brazil [11]. Given this historical influence, we believe that the relatively structured inclusion of mycology in Rio Grande do Sul may not reflect the educational reality in other Brazilian states, where exposure to fungal diseases in undergraduate curricula may be even more limited. We recognize that this strong local tradition may result in an overestimation of medical mycology education when compared to other Brazilian states.

Our findings reveal significant gaps in the teaching of medical mycology across most medical schools in Rio Grande do Sul, despite the region's historical contributions to the field. While core fungal infections are generally addressed, deficiencies in infrastructure, faculty training, and curricular innovation persist. The lack of institutional commitment and integration with reference centers highlights a broader need for national policies to promote fungal disease education. Expanding practical training, updating curricula in alignment with clinical guidelines, and investing in specialized faculty development will be essential to equip future physicians to manage the growing burden of fungal diseases in Brazil and beyond. To address these gaps, we propose developing standardized mycology curricula, increasing investment in laboratory infrastructure, promoting digital and wet-lab training, and establishing regional centers of excellence offering short-term fellowships or train-the-trainer programs. Proposals for teaching at universities and for training small and medium-sized laboratories across Brazil could be strengthened through comprehensive training programs developed in partnership with the government as well as with the private sector and nongovernmental organizations. These collaborative efforts would help expand access to high-quality training and foster innovation throughout the country.
